# Health Status of Female and Male Vegetarian and Vegan Endurance Runners Compared to Omnivores—Results from the NURMI Study (Step 2)

**DOI:** 10.3390/nu11010029

**Published:** 2018-12-22

**Authors:** Katharina Wirnitzer, Patrick Boldt, Christoph Lechleitner, Gerold Wirnitzer, Claus Leitzmann, Thomas Rosemann, Beat Knechtle

**Affiliations:** 1 Center for Research and Knowledge Management, Pedagogical University Tyrol, 6020 Innsbruck, Austria; 2 Department of Sport Science, University of Innsbruck, 6020 Innsbruck, Austria; 3 Faculty of Medicine, University of Gießen, 35390 Gießen, Germany; paboldt@aol.com; 4 ITEG, 6020 Innsbruck, Austria; christoph.lechleitner@iteg.at; 5 AdventureV & change2V, 6135 Stans, Austria; gerold@wirnitzer.at; 6 Institute of Nutrition, University of Gießen, 35390 Gießen, Germany; claus@leitzmann-giessen.de; 7 Institute of Primary Care, University of Zurich, 8091 Zurich, Switzerland; thomas.rosemann@usz.ch (T.R.); beat.knechtle@hispeed.ch (B.K.)

**Keywords:** vegetarian, vegan, half-marathon, marathon, running, health conscious, recreational athlete

## Abstract

Health effects of vegetarian and vegan diets are well known. However, data is sparse in terms of their appropriateness for the special nutritional demands of endurance runners. Therefore, the aim of this study was to investigate the health status of vegetarian (VER) and vegan endurance runners (VGR) and compare it to omnivorous endurance runners (OR). A total of 245 female and male recreational runners completed an online survey. Health status was assessed by measuring health-related indicators (body weight, mental health, chronic diseases, and hypersensitivity reactions, medication intake) and health-related behavior (smoking habits, supplement intake, food choice, healthcare utilization). Data analysis was performed by using non-parametric ANOVA and MANOVA. There were 109 OR, 45 VER and 91 VGR. Significant differences (*p* < 0.05) were determined for the following findings: (i) body weight for VER and VGR was less than for OR, (ii) VGR had highest *food choice* scores, and (iii) VGR reported the lowest prevalences of allergies. There was no association (*p* > 0.05) between diet and mental health, medication intake, smoking habits, supplement intake, and healthcare utilization. These findings support the notion that adhering to vegetarian kinds of diet, in particular to a vegan diet, is associated with a good health status and, thus, at least an equal alternative to an omnivorous diet for endurance runners.

## 1. Introduction

During an endurance event, such as a marathon running, body and mind are challenged to an extremely high degree. Athletes are exposed to several physiological and psychological challenges, in particular with regard to energy metabolism, body temperature and fluid balance [1,2,3]. A study by Hausswirth and Lehénaff highlighted the importance of fat metabolism, since an increase in free fatty acids and glycerol at the end of long-distance races crucially affects running economy and, thus, performance when the athlete is almost at the finish line [3]. Further important parameters with regard to running economy are maximal oxygen consumption, lactate-threshold, and metabolic efficacy [2]. Moreover, completing a long-distance race is a psychological challenge which requires favorable character traits, such as inhibitory control, the ability not only to inhibit motor response, but also to suppress processing of irrelevant information, and the ability to protect cognitive performance so that it is less influenced by emotional stimuli [1]. In order to meet all these requirements, a good health status and a strong mind are necessary and will contribute to good exercise performance [1,2].

An essential requirement for a good health status is the choice of an appropriate, healthy, and sustainable diet [4,5]. As endurance running is known as a kind of sport with high energy expenditure and, thus, consumption, an endurance athlete’s need for vitamins, trace elements and other valuable food ingredients besides macronutrient requirements is very high [4]. Therefore, a well-balanced energy turnover is crucial [4], resulting in the creation of a well-planned and reasonable nutrition strategy [5]. Current evidence suggests that one strategy could be adhering to a meatless diet rich in vegetables and fruits, such as a vegetarian kind of diet [6,7,8] (pp. 419–437). Vegetarian kind of diet is an umbrella term which subsumes four main dietary patterns: lacto-ovo-vegetarian, lacto-vegetarian, ovo-vegetarian, and vegan. Lacto-ovo vegetarians consume dairy products and eggs but no meat, poultry or seafood. Lacto-vegetarians eat dairy products but avoid eggs, meat, poultry, and seafood. Ovo-vegetarians eat eggs, but no dairy products, meat, poultry, or seafood. A vegan diet is characterized by the rejection of all products from animal sources, such as meat, fish/shellfish, milk and dairy products, eggs, and honey. A dietary pattern without any restriction is referred to as an omnivorous kind of diet [7].

Healthy vegetarian kinds of diet usually include complex carbohydrates, fiber, fruits, vegetables, and antioxidants [9]. Although potentially lower in some nutrients, such as zinc and vitamin B12 [9], carefully planned vegetarian kinds of diet meet or even exceed the nutritional requirements of athletes, in particular with regard to the intake of proteins, fatty acids and iron [6,7,8] (pp. 419–437). More than this, vegetarian kinds of diet are known to have further beneficial effects on health than just energy intake, in particular in terms of body weight control [10,11], the prevention of diabetes mellitus type 2 [12,13], ischemic heart disease [11,14], and protection against depression [15]. In addition, a vegetarian diet has been found to reduce the risk for some types of cancer, such as colon and prostate cancer [9,16]. Despite immediate health-related effects due to the consumption of healthy foods, being a vegetarian or vegan is often associated with a healthy lifestyle characterized by the avoidance of adverse health behavior, such as smoking and alcohol consumption, a high level of physical activity, and time for relaxation [8] (p. 393).

To date, little is known about the health status and health-related behavior of vegetarian and vegan endurance runners [17,18]. Most researchers have not classified their subjects by dietary subgroup [19]. Beyond that, these studies usually dealt with athletes in general, so that data in terms of endurance runners is sparse. A well-founded comparison of health characteristics between vegetarian, vegan and omnivorous endurance runners is lacking. Specific knowledge about the interconnectedness of diet choice and health could provide a better basis for athletes and their coaches, physicians, and nutritionists/dietitians, in order to optimize training and treatment strategies.

The aim of the study, therefore, was to investigate the health status of endurance runners and to compare athletes who adhere to a vegetarian or vegan diet to those who follow an omnivorous diet. Since a good state of health of non-active vegetarians and vegans is sound and compares favorably to that of omnivores [8] (p. 411), it was hypothesized that vegetarian and vegan endurance runners would have a better health status than omnivorous endurance runners.

## 2. Materials and Methods

### 2.1. Study Protocol and Ethics Approval

The study protocol [20] was approved by the ethics board of St. Gallen, Switzerland on 6 May 2015 (EKSG 14/145). The trial registration number is ISRCTN73074080.

### 2.2. Participants

The NURMI (Nutrition and Running High Mileage) Study was conducted in three steps following a cross-sectional design. Endurance runners, mainly from German-speaking countries including Germany, Austria, and Switzerland, were recruited. In addition, people from around the world were addressed. Participants were contacted mainly via social media, websites of the organizers of marathon events, online running communities, email lists, and runners’ magazines, as well as via magazines for health, vegetarian, and/or vegan nutrition and lifestyle, sports fairs, trade fairs on vegetarian and vegan nutrition and lifestyle, and through personal contacts. The characteristics of the subjects are presented in Table 1.

### 2.3. Procedures

#### 2.3.1. Experimental Approach

Participants completed an online survey within the NURMI Study Step 2, which was available in German and English at www.nurmi-study.com from 1 February 2015 to 31 December 2015. Prior to completing the questionnaires on physical and psychological health, participants were provided with a written description of the procedures and gave their informed consent to take part in the study.

**Scheme 1:**
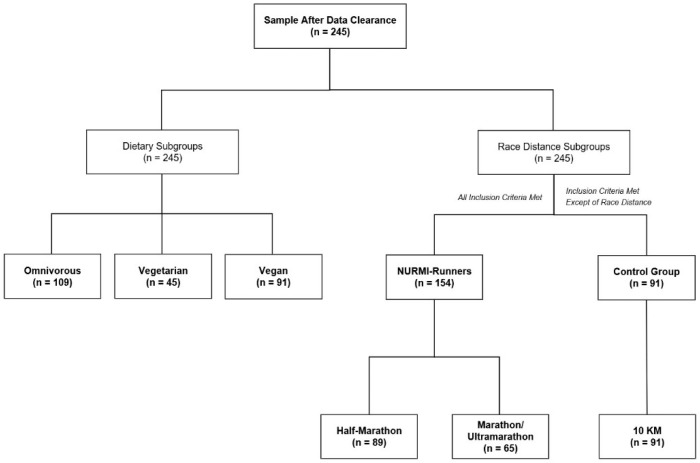
Categorization of participants.

For successful participation in the study, the following inclusion criteria were required: (1) written informed consent, (2) at least 18 years of age, (3) completed questionnaire, (4) successful participation in a running event of at least half-marathon distance in the past two years. Participants were classified into three dietary subgroups (Scheme 1): omnivorous (commonly known as Western diet, no dietary restrictions) diet; vegetarian (no meat); and vegan (no products from animal sources) [7]. In addition, they were categorized according to race distance: 10-km, half-marathon, and marathon/ultramarathon. Marathoners and ultramarathoners were pooled together since the marathon distance is included in an ultramarathon. A total of 91 highly-motivated runners provided accurate and useful answers with plenty of high-quality data. However, they had not successfully participated in either a half-marathon or marathon, but rather in a 10-km race. In order to avoid an irreversible loss of these valuable datasets, those who met all inclusion criteria but named a 10-km race as their running event were kept as the control group.

According to the WHO [21,22] the goal for individuals should be to maintain a BMI in the range 18.5–24.9 kg/m^2^ (BMI_NORM_) in order to achieve optimum health. They point to an increased risk of co-morbidities for a BMI 25.0–29.9 kg/m^2^, and moderate to severe risk of co-morbidities for a BMI > 30 kg/m^2^ [21,22]. Therefore, the calculated Body Mass Index (BMI_CALC_) was classified into three categories of body weight-to-height ratio (kg/m^2^): ≤ 18.49 < BMI_NORM_: 18.50–24.99 kg/m^2^ ≥ 25. Since the BMI of active runners could be below BMI_NORM_ [23], but in addition people with a higher BMI might start running in order to achieve and maintain a stable, healthy body weight, participants with BMI < 30 were included. BMI has been shown to be a significant performance-determining parameter for speed improvement in running over various distances, with a continuous increase in BMI from 19.57 (1.29) kg/m^2^ in marathoners to 23.3 (1.67) kg/m^2^ over the 100 m distance [24]. An optimal BMI for high running pace, reported for the best performers over 10 km and marathon distance, was found to be between 19–20 kg/m^2^.

#### 2.3.2. Data Clearance

In order to control for measures of (1) diet and (2) running, two groups of control questions were included, each within different sections of the survey. In order to control for a minimal status of health linked to a minimum level of fitness and to further enhance the reliability of datasets, the BMI approach following the WHO [21,22] was used. With a BMI ≥ 30 other health-protecting and/or weight loss strategies than running would be necessary to safely reduce body weight. Therefore, three participants with a BMI ≥ 30 were excluded from the data analysis.

**Scheme 2:**
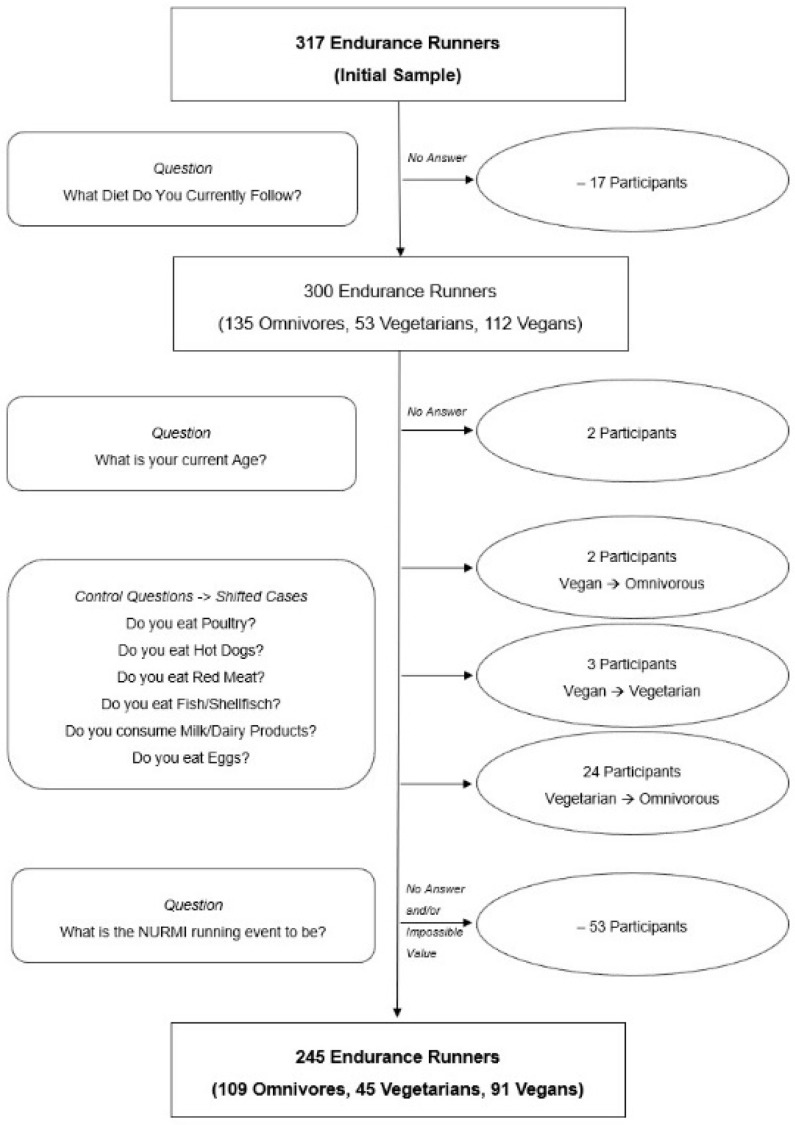
Flow chart of participants’ enrollment.

A total of 317 endurance runners completed the survey. Incomplete, inconsistent, and conflicting datasets were excluded from the data analysis. After data clearance a total of 245 runners with complete datasets were included for descriptive statistical analysis (Scheme 2).

### 2.4. Measures

Health status (latent variable) was derived by using both the two clusters ‘Health-related Indicators’ and ‘Health-related Behavior’. Each cluster pooled four dimensions, with each defined by specific items based on manifest measures. An overview of the variables is presented in Table 2.

The following health-related indicators described health outcomes: (1) body weight/BMI, (2) mental health (stress perception), (3) chronic diseases and hypersensitivity reactions: prevalence of chronic diseases (heart disease, state after heart attack, cancer), prevalence of metabolic diseases (diabetes mellitus 1, diabetes mellitus 2, hyperthyroidism, hypothyroidism), prevalence of hypersensitivity reactions (allergies, intolerances), and (4) medication intake (for thyroid disease, for hypertension, for cholesterol level, for contraception).

The following variables of health-related behavior described health outcomes: (1) smoking habits (current and former smoking), (2) supplement intake (supplements prescribed by a doctor, supplements for performance enhancement, supplements to cope with stress), (3) food choice (motivation, desired ingredients, avoided ingredients), and (4) healthcare utilization (regular check-ups). Resulting from this, eight domain scores (body weight/BMI, mental health, chronic diseases, and hypersensitivity reactions, medication intake, smoking, supplement intake, food choice, healthcare utilization) were derived, which generated scores between 0 and 1. Low scores indicate detrimental health effects, while higher scores indicate beneficial health effects (given as mean scores plus standard deviation, and percentage (%)).

### 2.5. Statistical Analysis

The statistical software R version 3.5.0 Core Team 2018 (R Foundation for Statistical Computing, Vienna, Austria) performed all statistical analyses. Exploratory analysis was performed by descriptive statistics (median and interquartile range (IQR)). Significant differences between dietary subgroups and domain scores to describe health status were calculated by using a non-parametric ANOVA. Chi-square test and Kruskal-Wallis test were used to examine the association between dietary subgroups and domain scores with nominal scale variables, and Wilcoxon test and Kruskal-Wallis test (ordinal and metric scale) approximated by using the F distributions.

*Statistical modeling*. State of health as the latent variable was derived by manifest variables (e.g., body weight, cancer, smoking, etc.). In order to scale the health status displayed by measures, items and dimensions, a heuristic index between 0 and 1 was defined (equivalence in all items). To test the statistical hypothesis considering significant differences between dietary subgroups, race distance and sex for each dimension a MANOVA was performed to define health status. The assumptions of the ANOVA were verified by residual analysis.

The level of statistical significance was set at *p* ≤ 0.05.

## 3. Results

A total of 317 endurance runners completed the survey, of whom 245 (141 women and 104 men) remained after data clearance with a mean age of 39 (IQR 17) years, from Germany (*n* = 177), Switzerland (*n* = 13), Austria (*n* = 44) and from other countries (*n* = 11; Belgium, Brazil, Canada, Italy, Luxemburg, Netherlands, Poland, Spain, UK).

A total of 109 participants followed an omnivorous diet, 45 reported to adhere to a vegetarian diet, and 91 to a vegan diet. In addition, there were a total of 91 10-km runners, 89 half-marathoners, and 65 marathoners/ultramarathoners.

### 3.1. Cluster ‘Health-Related Indicators’

#### 3.1.1. Dimension of Body Weight/BMI

**Figure 1:**
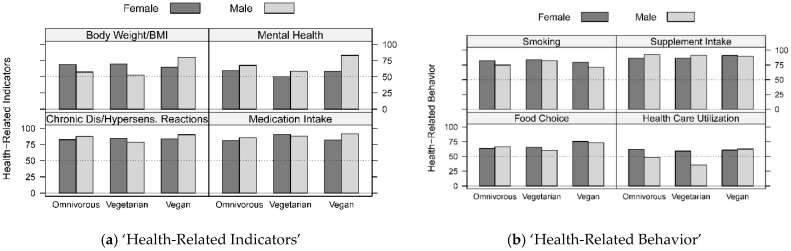
Indices of both clusters ‘Health-Related Indicators’ and ‘Health-Related Behavior’ of female and male endurance runners, displayed by dietary subgroups (as percentage, %). Low scores indicate detrimental health effects, high scores indicate beneficial health effects.

There was a significant difference in body weight between dietary subgroups (F_(2, 242)_ = 6.86, *p* = 0.001), with vegetarians and vegans showing lower body weight than omnivores. However, there was no difference in the health-related item BMI between dietary subgroups (*χ*^2^_(4)_ = 6.08, *p* = 0.193) (Table 3). Moreover, vegans had the highest counts for the health-related indicator *body weight/BMI* (0.69 (0.40), F_(2, 242)_ = 0.41, *p* = 0.662) (Figure 1a).

#### 3.1.2. Dimension of Mental Health

There was no significant association between diet group and stress perception (*χ*^2^_(2)_ = 1.78, *p* = 0.412) (Table 3). However, vegans had the highest score with regard to *mental health* (0.66 (0.48), F_(2, 219)_ = 0.88, *p* = 0.415) (Figure 1a).

#### 3.1.3. Dimension of Chronic Diseases and Hypersensitivity Reactions

There was no significant association between diet and the prevalence of cardiovascular diseases and cancer (*χ*^2^_(4)_ = 2.88, *p* = 0.578), and even between diet and prevalence of metabolic diseases (*χ*^2^_(10)_ = 7.14, *p* = 0.713). However, there was a significant difference between the prevalence of hypersensitivity reactions and diet (*χ*^2^_(4)_ = 12.87, *p* = 0.012), where vegan endurance runners stated least often that they had at least one allergy. In addition, omnivores reported having a food intolerance least often (Table 3). Omnivorous, vegan, and vegetarian runners scored similarly with regard to the health-related indicator *chronic diseases and hypersensitivity reactions* (respectively, 0.85 (0.20), 0.82 (0.20), and 0.85 (0.18), F_(2, 219)_ = 0.58, *p* = 0.562) (Figure 1a).

#### 3.1.4. Dimension of Medication Intake

There was no significant association between medication intake and dietary subgroup (*χ*^2^_(6)_ = 7.58, *p* = 0.271) (Table 3). Furthermore, there was no significant effect diet on the use of contraceptives (*χ*^2^_(2)_ = 0.70, *p* = 0.704). However, vegetarians had the highest scores with regard to medication intake, even though all dietary subgroups had similar scores (respectively, 0.84 (0.37), 0.90 (0.31), and 0.85 (0.36), F_(2, 219)_ = 0.41, *p* = 0.663) (Figure 1a).

### 3.2. Cluster ‘Health-Related Behavior’

#### 3.2.1. Dimension of Smoking Habits

Diet and current or former smoking were not significantly associated (*χ*^2^_(4)_ = 8.96, *p* = 0.062) (Table 4). Vegetarians showed the best health-related behavior with regard to *smoking habits* (0.83 (0.29), F_(2, 219)_ = 1.30, *p* = 0.275) (Figure 1b).

#### 3.2.2. Dimension of Supplement Intake

There was no significant association between diet and supplement intake prescribed by a doctor (*χ*^2^_(2)_ = 0.79, *p* = 0.675), the consumption of performance-enhancing substances (*χ*^2^_(4)_ = 4.09, *p* = 0.394) or the intake of substances to cope with stress (*χ*^2^_(4)_ = 1.79, *p* = 0.774) (Table 4). Vegans showed the best health-related behavior with regard to *supplement intake* (0.91 (0.19), F_(2, 219)_ = 0.35, *p* = 0.708) (Figure 1b).

#### 3.2.3. Dimension of Food Choice

There was no significant association between diet and food choice (i) because it is healthy (*χ*^2^_(2)_ = 1.59, *p* = 0.452) and health-promoting (*χ*^2^_(2)_ = 2.41, *p* = 0.300); or (ii) in order to obtain vitamins (*χ*^2^_(2)_ = 3.48, *p* = 0.175), minerals/trace elements (*χ*^2^_(2)_ = 0.56, *p* = 0.757), antioxidants (*χ*^2^_(2)_ = 4.25, *p* = 0.119) and fiber (*χ*^2^_(2)_ = 2.58, *p* = 0.276) (Table 4). Moreover, there was no significant association between diet and the avoidance of the following ingredients (Table 4): refined sugar (*χ*^2^_(2)_ = 3.95, *p* = 0.138), fat in general (*χ*^2^_(2)_ = 2.17, *p* = 0.339), white flour (*χ*^2^_(2)_ = 3.42, *p* = 0.181), sweets (*χ*^2^_(2)_ = 2.52, *p* = 0.284), nibbles (*χ*^2^_(2)_ = 0.15, *p* = 0.928), and alcohol (*χ*^2^_(2)_ = 0.29, *p* = 0.864).

However, there was a significant effect of diet on *food choice,* both (i) because it is good for maintaining health (*χ*^2^_(2)_ = 5.99, *p* = 0.050), with vegetarians and vegans reporting doing so more often; and (ii) in order to obtain phytochemicals (*χ*^2^_(2)_ = 9.93, *p* = 0.007), with vegans reporting doing so more often. Moreover, there was a significant association between diet and the avoidance of the following ingredients (Table 4): sweetener (*χ*^2^_(2)_ = 6.16, *p* = 0.046), saturated fats (*χ*^2^_(2)_ = 9.82, *p* = 0.007), cholesterol (*χ*^2^_(2)_ = 21.60, *p* < 0.001), and caffeine (*χ*^2^_(2)_ = 8.30, *p* = 0.016). Vegans were more likely to report considering avoiding these ingredients in their food choice than vegetarians and omnivores.

Vegan athletes had the highest scores in *food choice* compared to the other dietary subgroups (0.75 (0.20), F_(2, 219)_ = 6.76, *p* = 0.001) (Figure 1b).

#### 3.2.4. Dimension of Healthcare Utilization

There was no significant association between the use of regular health check-ups and diet (*χ*^2^_(2)_ = 1.91, *p* = 0.385) (Table 4). Vegan athletes had the highest scores with regard to *healthcare utilization* (0.61 (0.49), F_(2, 219)_ = 0.95, *p* = 0.389) (Figure 1b).

### 3.3. Results of the MANOVA

The findings of the MANOVA considering state of health are presented in Table 5, indicating significant differences (*p* < 0.05) for the following results: (i) race distance (F = 3.39, Df = 2, *p* = 0.036) and sex (F = 4.06, Df = 1, *p* = 0.045) had an effect on *mental health*, (ii) race distance had an impact on *chronic diseases* and *hypersensitivity reactions* (F = 3.27, Df = 2, *p* = 0.040), (iii) an association between sex and *smoking habits* (F = 4.22, Df = 1, *p* = 0.041), and (iv) an association between *food choice* and diet (F = 6.10, Df = 2, *p* = 0.003), with vegans having the highest scores (0.75).

However, the overall health status derived from all dimensions showed differences between race distances with statistical trend (F = 1.83, Df = 2, *p* = 0.71), but no significant differences were found for either diet or sex.

## 4. Discussion

This study intended to investigate the health status of vegetarian and vegan endurance runners and to compare it to omnivorous athletes, regarding potential differences in body weight, smoking habits, stress perception, the prevalence of chronic and metabolic diseases, the prevalence of allergies and food intolerances, medication and supplement intake, food choice, consumption of performance-enhancing substances, and healthcare utilization. In terms of assessing the state of health of endurance runners, it is generally accepted, that body weight, BMI and smoking behavior were known to affect running performance.

The main findings were: (i) vegetarians and vegans weighed significantly less than omnivores, (ii) vegans had the highest *food choice* scores, (iii) vegans reported choosing food because it is good for maintaining health more often, (iv) vegans reported avoiding sweeteners, saturated fats, cholesterol, and caffeine when choosing food more often, (v) vegans reported choosing food in order to obtain phytochemicals more often, and (vi) vegans reported the lowest prevalence of allergies.

### 4.1. Body Weight and BMI

A first important finding was that both vegetarians and vegans had lower body weight (62.00 kg (IQR 11.30) and 64.00 (IQR 10.00) kg, respectively) than omnivores (68.00 kg (IQR 16.70). At the same time, the majority of all participants had a BMI which was within the normal range of 18.50–24.99 kg/m^2^ (80 % in omnivores vs. 87 % in vegetarians vs. 82 % in vegans) [21,22,24], with vegans having the best *body weight/BMI* health scores.

BMI is a relevant parameter, since it is associated with an increased risk for diseases, such as cardiovascular diseases, if it is higher than BMI_NORM_, and with a couple of other disorders, such as anorexia nervosa, if it is below BMI_NORM_ [21,22]. In addition, it is a key factor with regard to running performance [24]. However, careful use and interpretation of the BMI is required. For example, the BMI of active runners could be below the normal range without being pathological [23].

In the light of this, the findings of the present study were in line with previous literature, where vegetarians and vegans also had lower BMI than meat-eaters [25,26,27]. Spencer et al. [28] attributed these differences in body weight and BMI mainly to differences in macronutrient intake between vegetarians, vegans, and omnivores. High protein and low fiber intakes were the factors most strongly associated with increasing BMI. Considering the fact that running speed and endurance performance are significantly associated with body mass and BMI [24], vegetarian kinds of diet are known to be a good basis for body weight control strategies for endurance athletes [7,16,29]. Meanwhile, athletes, as well as their coaches, have to be particularly aware of unintended body weight loss [30], which is why regular monitoring of body weight is recommended [27]. Beyond athletic concerns, vegetarian, but in particular vegan, dietary patterns are known as to be useful for body weight control for people who suffer from obesity and diabetes mellitus type 2 and hypercholesterinemia [12,13].

### 4.2. Vegetarians’ and Vegans’ Attitudes Towards Food Choice

While only the dimension *food choice* showed significant differences between dietary subgroups, overall the vegan dietary subgroup displayed the highest health scores from all dimensions (except for *medication intake*) and contributed to runners’ good state of health, ranging from 61%–91%.

A main result was that vegans showed the highest score (75%) in endurance runners in the dimension *food choice* to contribute most beneficially to the overall state of health. This means that they reported choosing food ingredients because they are good for maintaining health. This finding was consistent with available scientific literature.

Studies of vegetarians and vegans have identified a range of motivations for dietary choices [8] (p. 395), although personal health and animal welfare were predominant motives [31,32,33] (pp. 24–28). It has also been shown that vegetarians and vegans usually have healthier lifestyles than omnivores [8,34] (p. 393). Their healthy lifestyle is characterized by the avoidance of adverse health behaviors, such as smoking and alcohol consumption, a high level of physical activity, and time for relaxation. Moreover, vegetarians and vegans are usually well-educated, have a certain degree of intellectual curiosity, and are open to new experiences [8] (p. 393). These findings match the results from the present study and support the characterization of vegetarians, but vegans in particular, as being health-conscious. However, all participants, meaning vegetarian, vegan, and omnivorous endurance runners, reported health-reasons as being important for food and ingredients choice. This supports the notion that athletes in general are health-conscious [35], but vegan athletes are supposed to be those who care most about this specific health-related strategy [8] (p. 393).

However, there was no significant major effect of dietary subgroups on whether food or ingredients had been chosen because they were healthy or health-promoting, even though there was a slight predominance of vegetarian and vegan runners. This was not entirely in line with current scientific evidence, as it has been shown that vegetarians and vegans are usually more health-conscious than omnivores [33,34,36]. Notwithstanding this, the contradiction might be explained by the composition of the sample. As all participants were endurance runners, who are known to be health-conscious compared to non-active people of the general population [35], the predominance of vegetarians and vegans might have been compensated for in this regard. Furthermore, the survey was based on self-reporting, so the definition of what is healthy or health-promoting in terms of food ingredients would depend on individual definitions based on personal suggestions and beliefs. Therefore, the results might have been biased to a certain degree. However, as the majority of all runners reported considering health aspects when choosing food, the findings support the characterization of the participants as being health-conscious.

A further main result was that vegan participants reported choosing food ingredients in order to avoid cholesterol, caffeine, sweetener and saturated fats more often. This finding was in line with the literature as well [37] and supports the fact that vegans in particular are supposed to be health-conscious.

Even though caffeine and cholesterol do not have detrimental health effects or may even have beneficial health implications if they are consumed conscientiously [38,39], cholesterol, in particular, is believed to be a crucial factor in the genesis of cardiovascular diseases [39]. Cholesterol is known to be an important risk factor for cardiovascular disease due to the induction of the elevation of LDL levels. It has also been found that HDL levels, which protect against cardiovascular diseases, increase after cholesterol consumption, so moderate consumption has been recommended in some studies [39]. However, to date the interactions between cholesterol intake and LDL and HDL blood levels have not been revealed completely [40]. With regard to caffeine, moderate consumption can increase physical and mental performance, while excessive intake can induce abuse or dependence [39]. Thus, it seems likely that both substances can be consumed moderately without any severe harm. However, being aware of potential detrimental side effects and therefore conscientious consumption is recommended.

Consumption of a high number of saturated fats is associated with cardiovascular diseases, such as stroke, myocardial infarction, and hypertension [8] (p. 414). Since vegan diets are characterized by a low percentage of saturated fats and a high percentage of omega-3 and omega-6 fatty acids [41], adhering to a plant-based diet can be a good way to improve cardiovascular health.

Health-effects of artificial sweeteners are controversial. While a couple of these products, such as aspartame, have previously received a generally recognized status as being safe from the United States Food and Drug Administration, there is also evidence for detrimental effects, such as the manifestation of glucose intolerance, weight gain and triggering of migraine in susceptible individuals [42]). Moreover, carcinogen effects could not be ruled out yet [43]. Overall, avoiding these agents appears to be advisable, so that the fact that the vegan endurance runners of our sample reported avoiding ingestion of sweeteners characterized them as being particularly health-conscious once again.

In addition to the avoidance of harmful substances, such as cholesterol and saturated fats, vegans reported choosing food in order to obtain phytochemicals. This finding supports the fact that vegan athletes are particularly health-conscious, since the consumption of phytochemical-rich foods is an important benefit of any plant-based diet in that it might help to mitigate the effects of excess inflammation and to promote recovery from training [41].

### 4.3. Allergies and Food Intolerances

There was a significant association between the prevalence of hypersensitivity reactions and diet, whereby vegan endurance runners reported least often that they had at least one allergy (20% in vegans vs. 32% in omnivores and 36% in vegetarians). Among those vegan endurance runners, 10-km runners had the lowest prevalence of allergies. At the same time, omnivores reported having a food intolerance least often (1% in omnivores vs. 10% in vegetarians and 12% in vegans).

Current evidence is sparse in this regard. One study has detected higher allergy rates among vegetarians [44], whereas others found a protective effect of a diet rich in fruits and vegetables on the occurrence of allergic asthma [45] and food allergies [46,47]. However, a relatively high incidence of allergies in a sample of endurance runners is not unexpected. It is well known that endurance athletes are more likely to have allergies (prevalence up to 13%) than people from the general population (prevalence 7% to 8%) [48]. This is usually attributed to the amount of time runners spend outdoors, which is supposed to be associated with a drying of the airways and an increased exposure to airborne allergens [49]. In the light of this, the finding that the vegan 10-km runners reported the lowest prevalence of allergies appears to be plausible because they usually have to cope with smaller training volumes (daily and weekly mileage) to successfully compete over shorter race distances. As a consequence, these runners do not spent as much time outdoors as long-distance runners, such as half-marathoners and (ultra-)marathoners.

Regarding food intolerances, the current literature does not provide clear data in this regard. One study indicated that a vegan diet might beneficially affect the intestinal flora, which seems to lower the risk of irritable bowel disease [47], whereas another study identified a long-term vegetarian diet as being the reason for the occurrence of irritable bowel disease [50]. However, endurance athletes, in general, are supposed to be more susceptible to symptoms of food sensitivities, which can be similar to those of irritable bowel disease. Constant training challenges the bowel to an extreme degree and endurance running, in particular, might cause gastrointestinal complaints. Thus, the ability to cope with additional gastrointestinal stress induced by food intolerances would be reduced [51].

### 4.4. Stress Perception

There was no significant difference found between vegetarians, vegans, and omnivores in reported stress and perceived pressure. *Mental health* scores were high, regardless of diet choice. However, vegan endurance runners had the highest scores for *mental health*. These findings were in line with previous studies, which showed that both endurance running [52,53] and adhering to a vegan dietary pattern caused good mood states [54]. Certain characteristics of vegans, such as a high degree of health-awareness [8] (p. 393), and the beneficial effects of endurance running, such as relaxation due to physical activity and an increase in stress resilience [52,53], appear to be the key factors in this regard.

In the light of this, finding the optimal dose of endurance running appears to be relevant, since too little exercise does not lead to a reduction in stress, whilst too much exercise might even increase stress levels [52]. According to the findings of the present study, half-marathon running appears to be a good way to cope with stress. These findings (unpublished data from our laboratory) are discussed in detail elsewhere [55]. Moreover, among the participants of the present study, there was a slight male predominance among those runners who reported as not suffering from stress. This was in line with previous research where it was reported that male endurance athletes possess a slightly higher degree of mental toughness than their female counterparts, allowing them to cope better with stress during exercise and in everyday life [56].

### 4.5. Chronic Diseases

There was no significant differences between the dietary subgroups when considering heart disease requiring treatment, state after heart attack, cancer, diabetes mellitus type 1 and 2, hypothyroidism and hyperthyroidism. In addition, there was a low overall incidence of these diseases among our participants. The only exceptions seemed to be apparently higher rates of cancer and hypothyroidism among vegetarians and vegans, which could be explained by a statistical bias.

There were five females who had suffered from breast cancer. Three of them had decided to change their dietary habits in favor of a vegetarian kind of diet after diagnosis of cancer, which skewed the results. The higher prevalence of hypothyroidism could be explained by the female predominance among the vegetarian and vegan subjects, as it is well known that eight times as many women suffer from thyroid diseases in general, and in particular from hypothyroidism, than men [57].

The fact that there was no association between diet and the prevalence rates of chronic diseases partially contradicts the body of evidence. Adhering to a vegetarian or vegan diet is usually associated with a lower incidence of diabetes mellitus type 2 [7,12,13], hypothyroidism (Tonstad et al. 2013 [58]), coronary artery disease [11,14], depression [54] and obesity [11] compared to an omnivorous diet. However, this effect might be compensated for by the fact that all our subjects were endurance athletes, who are usually supposed to be health-conscious, especially compared to non-active people of the general population [35]. Furthermore, the mean age of our participants was quite low (43.00 ± 18.00 in omnivores, 39.00 ± 16.00 in vegetarians, and 37.00 ± 15.00 in vegans), so that it can be assumed that the peak age for the manifestation of most diseases had not been reached yet. Furthermore, the fact that people who suffer from severe diseases usually do not become endurance runners might have led to a certain decrease in prevalence rates as well.

### 4.6. Medication Intake

There was no significant association found between the intake of medication with diet. All subgroups had similar *medication intake* scores. As there was a low prevalence of chronic diseases among our subjects, it was not surprising that there was also a low number of athletes who had to take any medication on a regular basis. The only exceptions were the intake of hormones and medication for the thyroid. The relatively high number of athletes who take hormones could be explained by the use of contraceptive pills or other interventions among the female runners. With regard to thyroid medication, the relatively high incidence rates of hypothyroidism among the female subjects (8%) explains the number of subjects who were taking thyroid medication.

### 4.7. Smoking Habits

There was no significant association between diet and current or former smoking. Yet, a low rate of smokers in vegetarian, vegan and omnivorous runners was observed. Vegetarians had the best scores considering *smoking habits*. These findings were in line with previous research, which also showed low numbers of smokers among vegetarians and vegans [59,60]. Although the low rates among endurance runners could be explained by undesired performance limitations due to smoking [59], vegetarians and vegans are often particularly health-conscious and therefore the number of smokers would be quite low among them [8,60] (p. 393). In addition, we found that women were more likely to be non-smokers compared to men, which was in line with previous research [61]. Nonetheless, in the past years, the number of female smokers has increased, which is particularly displayed in the prevalence of smoking associated diseases, such as lung cancer [61].

### 4.8. Supplement Intake

The finding that percentages of supplement intake were similar in all diet groups is consistent with current evidence. At the same time, vegans had the highest *supplement intake* scores. These findings are in line with previous research which showed that vegetarian kinds of diet are not lacking in critical micronutrients and macronutrients, per se, but rather that nutrient deficits can occur in any kind of diet [62]. Plant-based diets, such as a vegan dietary pattern, are not worse in terms of daily nutrient intake than omnivorous kinds of diet [63]. A recent study showed that an omnivorous diet does not meet the required amount of intake of six nutrients on average (calcium, folate, magnesium, iron, copper, vitamin E), whereas in vegetarian diets the amount of the daily intake of three nutrients is too low on average (calcium, zinc, vitamin B12) [9]. Another study has even revealed higher diet quality scores in vegetarian runners than in non-vegetarian runners [17]. Thus, supplementation of certain nutrients can be recommended for omnivores, vegetarians, and vegans alike [63]. More than this, these findings underpinned the fact that vegans are particularly health-conscious, which has been confirmed in other studies as well [8,60] (p. 393).

The most frequently taken supplement mentioned by the participants was vitamin D. Vitamin D deficiency is usually not associated with a vegetarian or vegan diet [64], but is a common problem in the general population [65] and in particular among endurance runners. It was found that there is a very large difference between necessary and real intake in athletes, regardless of whether they adhere to a vegetarian, vegan, or omnivorous diet [66]. Thus, all endurance athletes have to be aware of vitamin D levels, irrespective of their dietary patterns.

### 4.9. Enhancement Substances

There was no significant association between dietary subgroups and the consumption of enhancement substances or anything to cope with stress. Vegans reported the lowest use of enhancement substances. As there was a low overall number of subjects who reported using such substances (*n* = 32 for the consumption of enhancement substances, *n* = 18 for the consumption of substances to cope with stress) it could be expected that the number among the dietary subgroups would be quite low as well. It is noteworthy that these findings contradicted a previous study by Wilson [18] who found that 40% of male marathon finishers reported the recent use of performance-enhancing supplements. However, since our subjects did (almost) not report using such substances, they are probably aware of the detrimental effects of substances to increase performance and would, therefore, have avoided intake. This applies especially to the vegan participants, who are known to be particularly health-conscious [8] (p. 393).

### 4.10. Healthcare Utilization

Vegans had the highest scores in *healthcare utilization*, although scores were similar for all dietary subgroups. Scientific data is sparse in this regard. In one study a higher need for healthcare has been found among vegetarians [44]. However, since our results showed a good state of health in vegan, vegetarian and omnivorous endurance runners, there seems to be no need for frequent doctor consultations. Furthermore, physical activity, such as endurance running, prevents diseases which could require consulting a doctor more frequently [52]. However, about half of the participants (54% in omnivores, 49% in vegetarians, and 61% in vegans) reported making use of routine health checks. Considering that the mean age of our participants was around 40 years, this was an encouraging result, as most health checks for the early recognition and treatment of severe diseases in Europe are recommended for people who are aged 40 years and older [67].

### 4.11. Limitations and Implications for Future Research

Some limitations of the study should be noted. The survey was based on self-reporting. Thus, the reliability of the data depended on the conscientiousness of the participants. However, this effect was controlled for diet, participation in running events and race distance by using control questions, each separated from the respective main question and included in different sections of the questionnaire. In addition, the small sample size and the pre-selection of the participants due to the fact that mainly highly motivated runners took part led to a lack of statistical representativeness which might have affected the results. Nonetheless, the high intrinsic motivation of the participants would also have led to an increase in the accuracy of their answers and thus to a high quality of the generated data. Moreover, it was striking that most subjects came from Germany (*n* = 177). This imbalance in the composition of the sample may have several causes. First, Germany has a population of 82 million, making it the largest German-speaking country [68]. As the core area of the present study were German-speaking countries, this predominance is displayed in the sample of the present study. Second, Germany has large vegetarian and vegan populations [69]. Since a couple of subjects were addressed via trade fairs on vegetarian and vegan nutrition and lifestyle, it was likely that the number of German participants would increase. Third, some of the largest running events, such as the Berlin Marathon [70], take place in Germany. Together, this might have led to an increase in German participants.

Nevertheless, the data contributes to the growing scientific interest in and research on vegetarianism and veganism as it relates to sports and exercise and can be taken as one step towards creating a broad body of evidence in this regard. Future studies should be performed on large randomized samples in order to improve statistical representativeness. Furthermore, measurement of the health status could be elaborated by including additional parameters, such as energy metabolism and fluid balance regulation. Thereby, the data generated from the participants’ self-report could be specified.

## 5. Conclusions

In summary, the findings revealed that all endurance runners had a good health status, regardless of the diet choice. At the same time, vegan athletes appeared to be extraordinarily health-conscious, in particular due to their food choice habits. These findings support the notion that adhering to vegetarian kinds of diet, but in particular to a vegan dietary pattern, is compatible with ambitious endurance running and can be an appropriate, at least equal and healthy alternative to an omnivorous diet for athletes.

## Figures and Tables

**Table 1:** Anthropometric and demographic characteristics of the subjects displayed by diet group.

		Omnivorous	Vegetarian	Vegan
**Number of Subjects**		100% (109)	100% (45)	100% (91)
**Sex**	Female	47% (51)	58% (26)	70% (64)
	Male	53% (58)	42% (19)	30% (27)
**Age (years) (median)**		43 (IQR 18)	39 (IQR 16)	37 (IQR 15)
**Body Weight (kg) (median)**		68 (IQR 16.7)	62 (IQR 11.3)	64 (IQR 10)
**BMI_CALC_ (kg/m^2^)**	≤18.49	4% (4)	7% (3)	9% (8)
	18.50–24.99	80% (87)	87% (39)	82% (75)
	≥25–29.99	17% (18)	7% (3)	9% (8)
**Race Distance**	10 km	34% (37)	33% (15)	43% (39)
	Half-marathon	36% (39)	44% (20)	33% (30)
	Marathon/Ultramarathon	30% (33)	22% (10)	24% (22)
**Academic Qualification**	No Qualification	0% (0)	0% (0)	1% (1)
	Upper Secondary Education/Technical Qualification/GCSE or Equivalent	38% (41)	38% (17)	27% (25)
	A Levels or Equivalent	24% (26)	16% (7)	22% (20)
	University Degree/Higher Degree (i.e., doctorate)	30% (33)	38% (17)	36% (33)
	No Answer	8% (9)	9% (4)	13% (12)
**Marital Status**	Divorced/Separated	3% (3)	4% (2)	11% (10)
	Married/Living with Partner	75% (82)	58% (26)	62% (56)
	Single	22% (24)	38% (17)	27% (25)
**Country of Residence**	Austria	21% (23)	18% (8)	14% (13)
	Germany	70% (76)	76% (34)	74% (67)
	Switzerland	7% (8)	4% (2)	3% (3)
	Other	2% (2)	2% (1)	9% (8)
**Motive for Diet Choice**	Health, wellbeing	81% (21)	85% (28)	90% (79)
	Sporting performance	54% (14)	33% (11)	59% (52)
	Food scandals	15% (4)	55% (18)	32% (28)
	Animal welfare	46% (12)	79% (26)	90% (79)
	Ecological aspects	50% (13)	76% (25)	83% (73)
	Social aspects	35% (9)	55% (18)	57% (50)
	Economic aspects	8% (2)	12% (4)	22% (19)
	Religion/spirituality	0% (0)	12% (4)	7% (6)
	Custom/tradition	15% (4)	0% (0)	2% (2)
	Taste/enjoyment	42% (11)	33% (11)	44% (39)
	No specific reason	4% (1)	0% (0)	0% (0)

10 km = 10-km control group. BMI_CALC_ = Body Mass Index (calculated). IQR = interquartile range.

**Table 2:** Overview of the variables in order to derive health status of endurance runners.

Cluster	Dimension	Indicator	Item	Measure
**Health-related Indicators**	**Body weight**/BMI	BMI_calc_^1^	Your current body weight (kg)?	Body weight (kg)
			Your height (m)?	Height (m)
	**Mental health**	Stress perception	Are you under pressure and/or are you suffering from stress?	Yes No
	**Chronic diseases and hypersensitivity reactions**	Cardiovascular diseases and Cancer	Are you currently suffering from the following chronic diseases or their direct consequences?	Heart disease requiring treatment Heart attack Cancer (now or in the past)?
		Metabolic diseases	Are you currently suffering from one of the following metabolic disease(s)?	Diabetes mellitus type 1 Diabetes mellitus type 2 Hyperthyroidism Hypothyroidism
		Hypersensitivity reactions	Are you currently suffering from ...?	Allergies Intolerances
	**Medication intake**		Do you take medicaments regularly (every day), for example, ...?	Thyroid High blood pressure Cholesterol and/or other blood serum lipid values contraceptive pill
**Health-related Behavior**	**Smoking habits**	Current consumption of cigarettes	Do you currently smoke?	Yes No
		Former consumption of cigarettes	Have you ever smoked?	Yes No
	**Supplement intake**	Substance intake for medical reasons	Do you take supplements prescribed by a doctor regularly (everyday)?	Yes No
		Intake of Performance enhancement substance	Do you take anything to boost your performance in your daily life, at work or while doing sport (e.g., energy drinks)?	Yes, regularly every day Yes, occasionally No
		Substance intake for stress coping	Do you take anything to help you cope with stress in your daily life, at work or while doing sport?	Yes, regularly every day Yes, occasionally No
	**Food choice**	Motivation for food choice	Do you choose ingredients and food on the basis Of the following (e.g., in view of the disease mentioned above or other illnesses)?	Healthy (e.g., if you are ill) Health-promoting (e.g., to prevent ill-health) Good for maintaining health (e.g., wholefoods)
		Avoided ingredients	Do you choose food in order to avoid particular ingredients or nutrients (e.g., in view of the diseases mentioned above or other illnesses or effects on health)?	Refined sugar Sweetener Fat in general Saturated fats Cholesterol Products made with white flour Sweet things (e.g., Jelly beans, chocolate drops, cream cakes) Nibbles (e.g., crisps, salted peanuts) Alcohol Caffeine or other stimulants (e.g., in coffee or energy drinks)
		Desired ingredients	Do you choose food because of particular valuable ingredients or nutrients (e.g., to prevent the diseases mentioned above or other illnesses or effects on health)?	Vitamins Minerals/trace elements Antioxidants Phytochemicals Fiber Other
	**Healthcare utilization**	Frequency of doctor consultations	How often have you seen a doctor in the last 12 months (except dentist and for routine check-ups)?	Never Once a month Every 2 months Every 3 months (four times a year) Every 6 months (twice a year) Once a year
		Utilization of regular health check-ups	Do you go for regular check-ups or routine health checks?	Yes No

^1^ BMI_CALC_ = Body Mass Index calculated.

**Table 3:** Descriptive results and ANOVA of the ‘Health-Related Indicators’ cluster.

Dimension	Omnivorous	Vegetarian	Vegan	Statistics
**Body Weight/BMI**				
Body Weight (kg) (median)	68.00 (IQR 16.70)	62.00 (IQR 11.30)	64.00 (IQR 10.00)	F_(2, 242)_ = 6.86, *p* = 0.001
BMI_CALC_				*χ*^2^_(4)_ = 6.08, *p* = 0.193
≤18.49	4% (4)	7% (3)	9% (8)	
18.50–24.99	80% (87)	87% (39)	82% (75)	
≥25–29.99	17% (18)	7% (3)	9% (8)	
**Mental Health**				*χ*^2^_(2)_ = 1.78, *p* = 0.412
Stress Perception				
Yes	36% (35)	46% (18)	34% (29)	
No	64% (63)	54% (21)	66% (56)	
**Chronic Diseases and Hypersensitivity Reactions**				
Prevalence of Chronic Diseases				*χ*^2^_(4)_ = 2.88, *p* = 0.578
Heart Disease	1% (1)			
Heart Attack				
Cancer			1% (1)	
No Diseases	99% (97)	100% (39)	99% (84)	
Prevalence of Metabolic Diseases				*χ*^2^_(10)_ = 7.14, *p* = 0.713
Diabetes Mellitus 1	1% (1)	3% (1)		
Diabetes Mellitus 2	2% (2)			
Hyperthyroidism	1% (1)	3% (1)	1% (1)	
Hypothyroidism	5% (5)	3% (1)	8% (7)	
Other Diseases	1% (1)		1% (1)	
No Diseases	90% (88)	92% (36)	89% (76)	
Prevalence of Hypersensitivity Reactions				*χ*^2^_(4)_ = 12.87, *p* = 0.012
Allergies	32% (31)	36% (14)	20% (17)	
Intolerances	1% (1)	10% (4)	12% (10)	
No Reactions	67% (66)	54% (21)	68% (58)	
**Medication Intake (regularly)**				*χ*^2^_(6)_ = 7.58, *p* = 0.271
Thyroid Disease	6% (6)	8% (3)	11% (9)	
Hypertension	5% (5)	3% (1)		
Cholesterol Level				
Other Medication	5% (5)		5% (4)	
No Medication	84% (82)	90% (35)	85% (72)	
Contraceptives	12% (12)	10% (4)	15% (13)	*χ*^2^_(2)_ = 0.70, *p* = 0.704

BMI_CALC_ = Body Mass Index (calculated). IQR = interquartile range.

**Table 4:** Descriptive results and ANOVA of the ‘Health-Related Behavior’ cluster.

Dimension	Omnivorous	Vegetarian	Vegan	Statistics
**Smoking Habits**				*χ*^2^_(4)_ = 8.96, *p* = 0.062
Non-Smoker	58% (57)	72% (28)	54% (46)	
Ex-Smoker	40% (39)	23% (9)	46% (39)	
Smoker	2% (2)	5% (2)		
**Supplement Intake**				
prescribed by doctor	8% (8)	10% (4)	6% (5)	*χ*^2^_(2)_ = 0.79, *p* = 0.675
to boost your performance (occasionally)	10% (10)	21% (8)	11% (9)	*χ*^2^_(4)_ = 4.09, *p* = 0.394
to boost your performance (regularly)	3% (3)	0 % (0)	2% (2)	
to cope with stress (occasionally)	5% (5)	5% (2)	8% (7)	*χ*^2^_(4)_ = 1.79, *p* = 0.774
to cope with stress (regularly)	2% (2)		2% (2)	
**Food Choice**				
Motivation				
because it is healthy	67% (66)	74% (29)	75% (64)	*χ*^2^_(2)_ = 1.59, *p* = 0.452
because it is health-promoting	81% (79)	79% (31)	88% (75)	*χ*^2^_(2)_ = 2.41, *p* = 0.300
because it is good for maintaining health	85% (83)	92% (36)	95% (81)	*χ*^2^_(2)_ = 5.99, *p* = 0.050
Avoided Ingredients				
Refined Sugar	62% (61)	56% (22)	73% (62)	*χ*^2^_(2)_ = 3.95, *p* = 0.138
Sweetener	74% (73)	59% (23)	80% (68)	*χ*^2^_(2)_ = 6.16, *p* = 0.046
Fat in General	39% (38)	46% (18)	49% (42)	*χ*^2^_(2)_ = 2.17, *p* = 0.339
Saturated Fats	53% (52)	46% (18)	72% (61)	*χ*^2^_(2)_ = 9.82, *p* = 0.007
Cholesterol	34% (33)	31% (12)	65% (55)	*χ*^2^_(2)_ = 21.60, *p* < 0.001
White Flour	64% (63)	59% (23)	74% (63)	*χ*^2^_(2)_ = 3.42, *p* = 0.181
Sweets	58% (57)	62% (24)	69% (59)	*χ*^2^_(2)_ = 2.52, *p* = 0.284
Nibbles	62% (61)	59% (23)	62% (53)	*χ*^2^_(2)_ = 0.15, *p* = 0.928
Alcohol	55% (54)	51% (20)	56% (48)	*χ*^2^_(2)_ = 0.29, *p* = 0.864
Caffeine	26% (25)	36% (14)	46% (39)	*χ*^2^_(2)_ = 8.30, *p* = 0.016
Desired Ingredients				
Vitamins	81% (79)	72% (28)	86% (73)	*χ*^2^_(2)_ = 3.48, *p* = 0.175
Minerals/Trace Elements	70% (69)	72% (28)	75% (64)	*χ*^2^_(2)_ = 0.56, *p* = 0.757
Antioxidants	47% (46)	44% (17)	60% (51)	*χ*^2^_(2)_ = 4.25, *p* = 0.119
Phytochemicals	42% (41)	31% (12)	59% (50)	*χ*^2^_(2)_ = 9.93, *p* = 0.007
Fiber	68% (67)	62% (24)	75% (64)	*χ*^2^_(2)_ = 2.58, *p* = 0.276
**Health Care Utilization**				
Regular check-ups or routine health checks	54% (53)	49% (19)	61% (52)	*χ*^2^_(2)_ = 1.91, *p*= 0.385

**Table 5:** Results of the MANOVA considering health status.

Cluster	Dimension	Subgroup	F	Df	*p*
**Health-related Indicators**	**Body weight/BMI**	Diet	0.75	2	0.475
		Race Distance	0.49	2	0.613
		Sex	0.62	1	0.432
	**Mental health**	Diet	0.91	2	0.402
		Race Distance	3.39	2	0.036
		Sex	4.06	1	0.045
	**Chronic diseases and hypersensitivity reactions**	Diet	0.49	2	0.611
		Race Distance	3.27	2	0.040
		Sex	0.77	1	0.381
	**Medication intake**	Diet	0.41	2	0.665
		Race Distance	0.15	2	0.859
		Sex	1.06	1	0.304
**Health-related Behavior**	**Smoking habits**	Diet	0.80	2	0.451
		Race Distance	1.78	2	0.172
		Sex	4.22	1	0.041
	**Supplement intake**	Diet	0.14	2	0.866
		Race Distance	0.93	2	0.395
		Sex	1.91	1	0.168
	**Food choice**	Diet	6.10	2	0.003
		Race Distance	1.11	2	0.331
		Sex	0.08	1	0.779
	**Healthcare utilization**	Diet	0.96	2	0.385
		Race Distance	1.52	2	0.222
		Sex	2.14	1	0.145

F = F-value. Df = Degrees of freedom. *p* = *p*-value for difference among groups.

## References

[1] Cona G., Cavazzana A., Paoli A., Marcolin G., Grainer A., Bisiacchi P.S. (2015). It’s a matter of mind! Cognitive functioning predicts the athletic performance in ultra-marathon runners. PLoS ONE.

[2] Joyner M.J., Coyle E.F. (2008). Endurance exercise performance: The physiology of champions. J. Physiol..

[3] Hausswirth C., Lehénaff D. (2001). Physiological demands of running during long distance runs and triathlons. Sports Med..

[4] Deldicque L., Francaux M. (2015). Recommendations for healthy nutrition in female endurance runners: An update. Front. Nutr..

[5] Ormsbee M.J., Bach C.W., Baur D.A. (2014). Pre-exercise nutrition: The role of macronutrients, modified starches and supplements on metabolism and endurance performance. Nutrients.

[6] Fuhrman J., Ferreri D.M. (2010). Fueling the vegetarian (vegan) athlete. Curr. Sports Med. Rep..

[7] Melina V., Craig W., Levin S. (2016). Position of the academy of nutrition and dietetics: Vegetarian diets. J. Acad. Nutr. Diet..

[8] Wirnitzer K.C., Grumezescu A.M., Holban A.M. (2018). Vegan nutrition: Latest boom in health and exercise. Therapeutic, Probiotic, and Unconventional Foods. Section 3: Unconventional Foods and Food Ingredients.

[9] Turner D.R., Sinclair W.H., Knez W.L. (2014). Nutritional adequacy of vegetarian and omnivore dietary intakes. J. Nutr. Health Sci..

[10] Berkow S.E., Barnard N. (2006). Vegetarian diets and weight status. Nutr. Rev..

[11] Kahleova H., Levin S., Barnard N.D. (2018). Vegetarian dietary patterns and cardiovascular disease. Prog. Cardiovasc. Dis..

[12] Kahleova H., Pelikanova T. (2015). Vegetarian diets in the prevention and treatment of type 2 diabetes. J. Am. Coll. Nutr..

[13] Kahleova H., Levin S., Barnard N. (2017). Cardio-metabolic benefits of plant-based diets. Nutrients.

[14] Tuso P., Stoll S.R., Li W.W. (2015). A plant-based diet, atherogenesis, and coronary artery disease prevention. Perm J..

[15] Liu X., Yan Y., Li F., Zhang D. (2016). Fruit and vegetable consumption and the risk of depression: A meta-analysis. Nutrition.

[16] Fraser G.E. (1999). Associations between diet and cancer, ischemic heart disease, and all-cause mortality in non-Hispanic white California Seventh-day Adventists. Am. J. Clin. Nutr..

[17] Turner-McGrievy G.M., Moore W.J., Barr-Anderson D. (2016). The interconnectedness of diet choice and distance running: Results of the research understanding the nutrition of endurance runners (runner) study. Int. J. Sport Nutr. Exerc. Metab..

[18] Wilson P.B. (2016). Nutrition behaviors, perceptions, and belief of recent marathon finishers. Phys. Sportsmed..

[19] Diehl K., Thiel A., Zipfel S., Mayer J., Litaker D.G., Schneider S. (2012). How healthy is the behavior of young athletes? A systematic literature review and meta-analyses. J. Sports Sci. Med..

[20] Wirnitzer K., Seyfart T., Leitzmann C., Keller M., Wirnitzer G., Lechleitner C., Rüst C.A., Rosemann T., Knechtle B. (2016). Prevalence in running events and running performance of endurance runners following a vegetarian or vegan diet compared to non-vegetarian endurance runners: The NURMI Study. SpringerPlus.

[21] Word Health Organization (WHO) (2018). WHO Regional Office for Europe. Body Mass Index—BMI. http://www.euro.who.int/en/health-topics/disease-prevention/nutrition/a-healthy-lifestyle/body-mass-index-bmi.

[22] Word Health Organization (WHO) (2018). Global Health Observatory (GHO) Data. Mean Body Mass Index (BMI). Situation and Trends. http://www.who.int/gho/ncd/risk_factors/bmi_text/en/.

[23] Marc A., Sedeaud A., Guillaume M., Rizk M., Schipman J., Antero-Jacquemin J., Haida A., Berthelot G., Toussaint J.F. (2014). Marathon progress: Demography, morphology and environment. J. Sports Sci..

[24] Sedeaud A., Marc A., Marck A., Dor F., Schipman J., Dorsey M., Haida A., Berthelot G., Toussaint J.F. (2014). BMI, a performance parameter for speed improvement. PLoS ONE.

[25] Barnard N.D., Levin S.M., Yokoyama Y. (2015). A systematic review and meta-analysis of changes in body weight in clinical trials of vegetarian diets. J. Acad. Nutr. Diet..

[26] Clarys P., Deliens T., Huybrechts I., Deriemaeker P., Vanaelst B., De Keyzer W., Hebbelinck M., Mullie P. (2014). Comparison of nutritional quality of the vegan, vegetarian, semi-vegetarian, pesco-vegetarian and omnivorous diet. Nutrients.

[27] Venderley A.M., Campbell W.W. (2006). Vegetarian diets: Nutritional considerations for athletes. Sports Med..

[28] Spencer E.A., Appleby P.N., Davey G.K., Key T.J. (2003). Diet and body mass index in 38000 EPIC-Oxford meat-eaters, fish-eaters, vegetarians and vegans. Int. J. Obes. Relat. Metab. Disord..

[29] Appleby P.N., Thorogood M., Mann J.I., Key T.J. (1999). The Oxford Vegetarian Study: An overview. Am. J. Clin. Nutr..

[30] Barr S.I., Rideout C.A. (2004). Nutritional considerations for vegetarian athletes. Nutrition.

[31] Fox N., Ward K.J. (2008). You are what you eat? Vegetarianism, health and identity. Soc. Sci. Med..

[32] Leitzmann C., Keller M. (2013). Vegetarische Ernährung.

[33] Waldmann A., Koschizke J.W., Leitzmann C., Hahn A. (2003). Dietary intakes and lifestyle factors of a vegan population in Germany: Results from the German Vegan Study. Eur. J. Clin. Nutr..

[34] Bedford J.L., Barr S.I. (2005). Diets and selected lifestyle practices of self-defined adult vegetarians from a population-based sample suggest they are more “health conscious”. Int. J. Behav. Nutr. Phys. Act..

[35] Pate R.R., Trost S.G., Levin S., Dowda M. (2000). Sports participation and health-related behaviors among us youth. Arch. Pediatr. Adolesc. Med..

[36] Chang-Claude J., Hermann S., Eilber U., Steindorf K. (2005). Lifestyle determinants and mortality in German vegetarians and health-conscious persons: Results of a 21-year follow-up. Cancer Epidemiol. Biomark. Prev..

[37] Nieman D.C., Underwood B.C., Sherman K.M., Arabatzis K., Barbosa J.C., Johnson M., Shultz T.D. (1989). Dietary status of Seventh-Day Adventist vegetarian and non-vegetarian elderly women. J. Am. Diet. Assoc..

[38] Cappelletti S., Piacentino D., Sani G., Aromatario M. (2015). Caffeine: Cognitive and physical performance enhancer or psychoactive drug?. Curr. Neuropharmacol..

[39] Fernandez M.L. (2012). Rethinking dietary cholesterol. Curr. Opin. Clin. Nutr. Metab. Care.

[40] Berger S., Raman G., Vishwanathan R., Jacques P.F., Johnson E.J. (2015). Dietary cholesterol and cardiovascular disease: A systematic review and meta-analysis. Am. J. Clin. Nutr..

[41] Rogerson D. (2017). Vegan diets: Practical advice for athletes and exercisers. J. Int. Soc. Sports Nutr..

[42] Sharma A., Amarnath S., Thulasimani M., Ramaswamy S. (2016). Artificial sweeteners as a sugar substitute: Are they really safe?. Indian J. Pharmacol..

[43] Soffritti M., Padovani M., Tibaldi E., Falcioni L., Manservisi F., Belpoggi F. (2014). The carcinogenic effects of aspartame: The urgent need for regulatory re-evaluation. Am. J. Ind. Med..

[44] Burkert N.T., Muckenhuber J., Großschädl F., Rásky É., Freidl W. (2014). Nutrition and health—The association between eating behavior and various health parameters: A matched sample study. PLoS ONE.

[45] Romieu I., Varraso R., Avenel V., Leynaert B., Kauffmann F., Clavel-Chapelon F. (2006). Fruit and vegetable intakes and asthma in the E3N study. Thorax.

[46] Du Toit G., Tsakok T., Lack S., Lack G. (2016). Prevention of food allergy. J. Allergy Clin. Immunol..

[47] Glick-Bauer M., Yeh M.-C. (2014). The health advantage of a vegan diet: Exploring the gut microbiota connection. Nutrients.

[48] Van der Wall E.E. (2014). Long-distance running: Running for a long life?. Neth. Heart J..

[49] Hoffman M.D., Krishnan E. (2014). Health and exercise-related medical issues among 1,212 ultramarathon runners: Baseline findings from the Ultrarunners longitudinal tracking (ULTRA) study. PLoS ONE.

[50] Buscail C., Sabate J.-M., Bouchoucha M., Torres M.J., Allès B., Hercberg S., Benamouzig R., Julia C. (2017). Association between self-reported vegetarian diet and the irritable bowel syndrome in the French NutriNet cohort. PLoS ONE.

[51] Miall A., Khoo A., Rauch C., Snipe R.M.J., Camões-Costa V.L., Gibson P.R., Costa R.J.S. (2018). Two weeks of repetitive gut-challenge reduce exercise-associated gastrointestinal symptoms and malabsorption. Scand. J. Med. Sci. Sports.

[52] Shipway R., Holloway I. (2010). Running free: Embracing a healthy lifestyle through distance running. Perspect. Public Health.

[53] Knechtle B., Quarella A. (2007). Running helps—Or how you escape depression without a psychiatrist and end up running a marathon!. Praxis (Bern 1994).

[54] Beezhold B., Radnitz C., Rinne A., DiMatteo J. (2015). Vegans report less stress and anxiety than omnivores. Nutr. Neurosci..

[55] Boldt P., Knechtle B., Nikolaidis P., Lechleitner C., Wirnitzer G., Leitzmann C., Rosemann T., Wirnitzer K. Half-Marathoners Report Best Health Status—Results from the NURMI Study (Step 2). Unpublished data from our laboratory. Eur. J. Sports Sci.

[56] Zeiger J.S., Zeiger R.S. (2018). Mental toughness latent profiles in endurance athletes. PLoS ONE.

[57] Dunn D., Turner C. (2016). Hypothyroidism in women. Nurs. Womens Health.

[58] Tonstad S., Nathan E., Oda K., Fraser G. (2013). Vegan diets and hypothyroidism. Nutrients.

[59] Marti B., Abelin T., Minder C.E., Vader J.P. (1988). Smoking, alcohol consumption, and endurance capacity: An analysis of 6,500 19-year-old conscripts and 4,100 joggers. Prev. Med..

[60] Appleby P.N., Crowe F.L., Bradbury K.E., Travis R.C., Key T.J. (2016). Mortality in vegetarians and comparable nonvegetarians in the United Kingdom. Am. J. Clin. Nutr..

[61] Peters S.A., Huxley R.R., Woodward M. (2014). Do smoking habits differ between women and men in contemporary Western populations? Evidence from half a million people in the UK Biobank study. BMJ Open.

[62] Schüpbach R., Wegmüller R., Berguerand C., Bui M., Herter-Aeberli I. (2017). Micronutrient status and intake in omnivores, vegetarians and vegans in Switzerland. Eur. J. Nutr..

[63] McDougall C., McDougall J. (2013). Plant-Based Diets Are Not Nutritionally Deficient. Perm J..

[64] Baig J.A., Sheikh S.A., Islam I., Kumar M. (2013). Vitamin D status among vegetarians and non-vegetarians. J. Ayub Med. Coll. Abbottabad.

[65] Gani L.U., How C.H. (2015). Vitamin D deficiency. Singap. Med. J..

[66] Larson-Meyer E. (2013). Vitamin D supplementation in athletes. Nestle Nutr. Inst. Workshop Ser..

[67] Schülein S., Taylor K.J., Schriefer D., Blettner M., Klug S.J. (2017). Participation in preventive health check-ups among 19,351 women in Germany. Prev. Med. Rep..

[68] Ehling M., Pötzsch O. (2010). Demographic changes in Germany up to 2060—Consequences for blood donation. Transfus. Med. Hemother..

[69] Leitzmann C. (2014). Vegetarian nutrition: Past, present, future. Am. J. Clin. Nutr..

[70] Haeusler K.G., Herm J., Kunze C., Krüll M., Brechtel L., Lock J., Hohenhaus M., Heuschmann P.U., Fiebach J.B., Haverkamp W. (2012). Rate of cardiac arrhythmias and silent brain lesions in experienced marathon runners: Rationale, design and baseline data of the Berlin Beat of Running study. BMC Cardiovasc. Disord..

